# Serum Parathyroid Hormone and Long-Term Mortality in Hospitalized Patients: A Real-World Cohort Study

**DOI:** 10.3390/jcm15062272

**Published:** 2026-03-17

**Authors:** Hüseyin Öztürk, Ece Çiftçi Öztürk, Özge Yasun, Esra Kaplan Arat, Onur Arat, Seher İrem Şahin, Büşra Çetintulum Aydın, Hayriye Esra Ataoğlu

**Affiliations:** 1 Department of Internal Medicine, Başakşehir Çam and Sakura City Hospital, University of Health Science, İstanbul 34480, Turkey; huseyinozturkdr@gmail.com (H.Ö.); ozgeyasun@gmail.com (Ö.Y.); 2 Department of Internal Medicine, Haseki Traning and Research Hospital, University of Health Science, İstanbul 34275, Turkey; siremcetin@gmail.com (S.İ.Ş.); busracetintulum@hotmail.com (B.Ç.A.); eataoglu@gmail.com (H.E.A.); 3 Department of Internal Medicine, Izmit Seka State Hospital, Kocaeli 41100, Turkey; esra15esra@gmail.com (E.K.A.); onur135596@gmail.com (O.A.)

**Keywords:** parathyroid hormone, mortality, prognosis, hyperparathyroidism

## Abstract

**Background:** Parathyroid hormone (PTH) is closely linked to mineral metabolism, kidney function, inflammation, and cardiovascular disease. However, its clinical significance in unselected hospitalized populations remains insufficiently characterized. We aimed to evaluate the prevalence of elevated PTH and its association with long-term mortality in a real-world cohort of internal medicine inpatients. **Methods:** In this retrospective cohort study, electronic records of adults hospitalized in an internal medicine department in 2019 were reviewed. Patients with available in-hospital PTH measurements were included. Elevated PTH was defined as >88 pg/mL. Clinical characteristics and laboratory parameters were recorded. The primary outcome was all-cause mortality with a minimum follow-up of 2 years. Cox proportional hazards models adjusted for clinically relevant covariates were used to examine the association between PTH and mortality. **Results:** A total of 1595 patients were included, of whom 567 (35.5%) had elevated PTH levels. Patients with elevated PTH were older and had a higher burden of chronic kidney disease, cardiovascular disease, and inflammatory and nutritional abnormalities. During a mean follow-up of 22 ± 13 months, mortality occurred more frequently in patients with elevated PTH than in those with normal levels (56.1% vs. 33.7%, *p* < 0.001). After multivariable adjustment, elevated PTH remained independently associated with increased mortality risk-adjusted HR (1.36, 95% CI 1.15–1.62, *p* < 0.001). **Conclusions:** Elevated PTH is common among hospitalized internal medicine patients and is associated with increased long-term mortality. PTH may represent an integrated biomarker reflecting disease burden, inflammation, and renal dysfunction and could contribute to risk stratification in hospitalized populations. Prospective studies are warranted to clarify underlying mechanisms and clinical implications.

## 1. Introduction

Parathyroid hormone (PTH) is a central regulator of calcium–phosphate homeostasis and plays a fundamental role in bone and mineral metabolism. PTH secretion is tightly regulated by plasma calcium, phosphate, calcitriol, and fibroblast growth factor-23, with calcium acting as the primary determinant. Even small decreases in serum calcium concentration rapidly stimulate PTH release, leading to increased bone resorption, enhanced renal tubular calcium reabsorption, reduced phosphate reabsorption, and activation of renal 1α-hydroxylase in the proximal tubules. This enzymatic activation increases the conversion of 25-hydroxyvitamin D to its biologically active form, 1,25-dihydroxyvitamin D (calcitriol), thereby promoting intestinal calcium absorption [1].

Beyond its classical endocrine functions, accumulating evidence suggests that PTH is associated with cardiovascular disease and adverse clinical outcomes. Elevated PTH levels have been linked to increased hospitalization in heart failure and higher risks of cardiovascular and all-cause mortality [2]. In patients with chronic kidney disease (CKD), secondary hyperparathyroidism is common and has been implicated in the development of hypertension, left ventricular hypertrophy, and vascular and valvular calcification [3,4].

Most previous studies evaluating the prognostic significance of PTH have focused on community-based populations or patients with CKD. Large observational cohorts and meta-analyses have demonstrated an association between elevated PTH levels and increased mortality risk. More recent evidence further suggests that this relationship may not be strictly linear, with several studies suggesting a potential J-shaped association between PTH levels and adverse outcomes. However, hospitalized internal medicine patients represent a distinct and clinically vulnerable population characterized by multimorbidity, acute illness, systemic inflammation, and metabolic disturbances. Data regarding the prevalence and prognostic implications of elevated PTH in this setting remain limited.

In addition to its role in mineral metabolism, PTH has increasingly been recognized as a marker of systemic disease burden. Several studies have suggested that elevated PTH levels may reflect complex interactions between renal dysfunction, inflammation, nutritional status, and cardiovascular risk. Hospitalized patients represent a particularly vulnerable population with multimorbidity and acute illness. In this context, biomarkers that integrate multiple physiological pathways may provide valuable information for clinical risk stratification. However, the clinical relevance of elevated PTH levels in unselected hospitalized internal medicine populations has not been sufficiently explored.

Therefore, we aimed to determine the prevalence of elevated PTH among hospitalized internal medicine patients and investigate its independent association with long-term all-cause mortality.

## 2. Methods

### 2.1. Study Design and Population

This retrospective cohort study was conducted at the Internal Medicine Department of Haseki Training and Research Hospital after approval from the Institutional Clinical Research Ethics Committee (approval number: 2021-135). This study was performed in accordance with the principles of the Declaration of Helsinki. Electronic medical records of all adult patients hospitalized between 1 January and 31 December 2019 were screened (*n* = 3850). Patients were eligible if serum parathyroid hormone (PTH) levels had been measured during hospitalization as part of routine clinical evaluation.

Exclusion criteria were:

- age < 18 years
- hospital stay < 24 h
- repeated hospitalizations (only the first admission was included)
- missing key clinical or laboratory data
- PTH levels below the lower reference limit (<12 pg/mL)

The institutional laboratory reference range for PTH was 12–88 pg/mL, which corresponds to the reference interval validated for the Roche Cobas 8000 electrochemiluminescence immunoassay used in our laboratory. According to this reference range, patients were categorized into two groups:

- Elevated PTH: >88 pg/mL
- Normal PTH: 12–88 pg/mL

**Figure 1:**
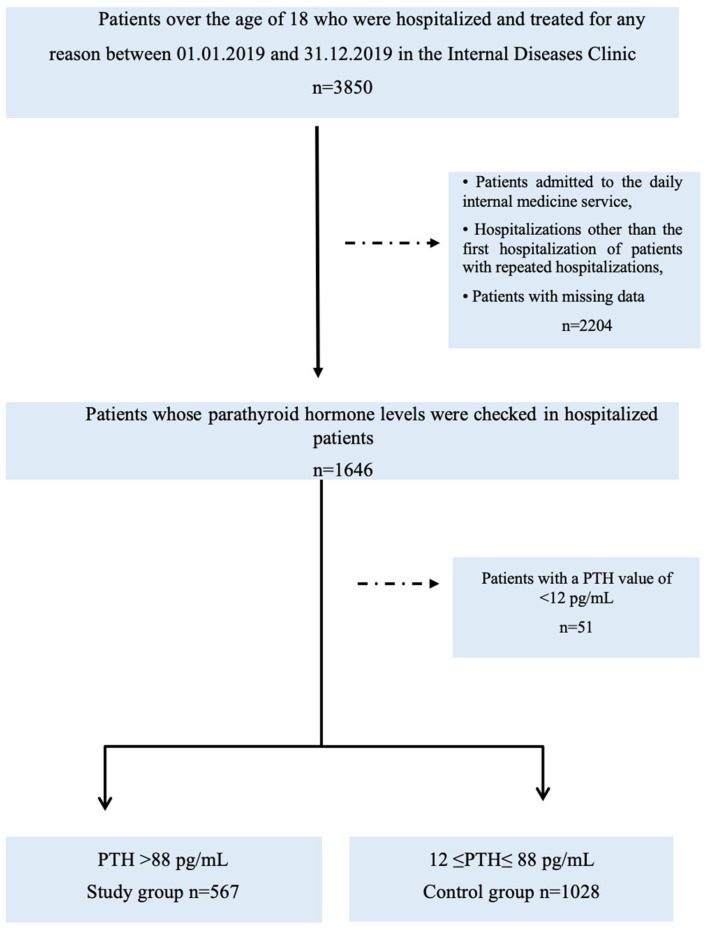
Study Flow Chart.

After applying the exclusion criteria, the final study cohort consisted of 1595 patients. This cohort reflects a real-world population of hospitalized patients in whom PTH testing was clinically indicated (Figure 1). This study reflects real-world clinical practice in hospitalized patients in whom PTH testing was performed based on clinical indications.

### 2.2. Laboratory Measurements

Blood samples were obtained in the morning after overnight fasting according to standard hospital protocols. Biochemical measurements, including PTH, were performed using the Cobas 8000 analyzer (Roche Diagnostics, Mannheim, Germany) based on an electrochemiluminescence immunoassay method. Hematological parameters were measured using the CAL 8000 system (Mindray Bio-Medical Electronics Co., Shenzhen, China). Internal quality control procedures were performed regularly according to manufacturer recommendations, and the laboratory participated in external quality assessment programs to ensure analytical reliability. Laboratory variables included markers reflecting renal function, inflammation, nutritional status, and mineral metabolism:

- hemoglobin, leukocyte, neutrophil, lymphocyte, and platelet counts
- C-reactive protein and procalcitonin
- creatinine, liver enzymes, and lipid profile
- HbA1c and thyroid-stimulating hormone
- 25-hydroxyvitamin D
- albumin
- calcium, phosphorus, and magnesium

### 2.3. Outcome Definition and Follow-Up

The primary outcome of this study was all-cause mortality. Mortality data were obtained from the Hospital Information Management System. Follow-up time was calculated from the date of hospital admission (index date) until the date of death or the last available record. Patients were followed for a minimum of two years after the last included hospitalization. Mortality data were unavailable for 91 patients; these individuals were censored at the date of the last recorded hospital contact.

### 2.4. Covariates

Demographic characteristics, comorbidities, length of hospitalization, and laboratory parameters measured at admission were extracted from electronic medical records. Recorded comorbidities included:

- diabetes mellitus
- hypertension
- ischemic heart disease
- chronic kidney disease
- thyroid disease
- solid malignancy
- hematologic malignancy

These variables were selected based on clinical relevance and the previous literature evaluating mortality risk in hospitalized populations.

### 2.5. Statistical Analysis

Continuous variables were evaluated for normality using graphical inspection (histograms and Q–Q plots) and the Kolmogorov–Smirnov test. Normally distributed variables are presented as mean ± standard deviation, whereas non-normally distributed variables are expressed as median and interquartile range (IQR). Categorical variables are presented as counts and percentages. Between-group comparisons were performed using:

- Student’s *t*-test or Welch’s *t*-test for normally distributed variables
- Mann–Whitney U test for non-normally distributed variables
- Chi-square test for categorical variables

Survival probabilities were estimated using the Kaplan–Meier method, and differences between groups were evaluated using the log-rank test. Survival analysis accounted for censored observations, representing patients who were alive at the end of follow-up or lost to follow-up during the study period. The association between PTH levels and mortality was assessed using Cox proportional hazards regression models. Clinically relevant covariates were selected a priori and included in the multivariable models to account for potential confounding. These included:

- age (continuous variable)
- sex (binary variable)
- comorbidities (binary variables)
- renal function (serum creatinine)
- inflammatory markers (CRP, procalcitonin)
- nutritional markers (albumin and hemoglobin)

Results are presented as hazard ratios (HRs) with 95% confidence intervals (CIs). A two-sided *p*-value < 0.05 was considered statistically significant. All statistical analyses were performed using IBM SPSS Statistics for Windows, version 22.0 (IBM Corp., Armonk, NY, USA).

## 3. Results

A total of 1595 hospitalized patients with available PTH measurements were included in the analysis. Elevated PTH levels (>88 pg/mL) were present in 567 patients (35.5%), while 1028 patients (64.5%) had PTH levels within the reference range. Patients with elevated PTH were significantly older and more frequently female compared with those with normal PTH levels (Table 1). In addition, the elevated PTH group had a higher burden of comorbidities, particularly hypertension, ischemic heart disease, and chronic kidney disease (all *p* < 0.001). Regarding laboratory parameters, patients with elevated PTH had lower hemoglobin, platelet, lymphocyte, albumin, calcium, and 25-hydroxyvitamin D levels, whereas creatinine, phosphorus, alkaline phosphatase, neutrophil count, and CRP levels were significantly higher compared with patients with normal PTH levels (Table 1). These findings indicate a greater burden of renal dysfunction, systemic inflammation, and impaired nutritional status in patients with elevated PTH.

Follow-up data were available for 1504 patients, with a mean follow-up duration of 22 ± 13 months. During follow-up, mortality occurred significantly more frequently in patients with elevated PTH compared with those with normal PTH levels (56.1% vs. 33.7%, *p* < 0.001). Patients who died during follow-up were older and exhibited higher levels of inflammatory markers, creatinine, phosphorus, and liver enzymes, along with lower hemoglobin, lymphocyte, platelet, and albumin levels compared with survivors. Additionally, hypertension, ischemic heart disease, chronic kidney disease, and malignancy were more prevalent among non-survivors (Table 2).

Kaplan–Meier survival analysis demonstrated significantly lower cumulative survival in patients with elevated PTH levels compared with those with normal PTH levels (log-rank *p* < 0.001).

Cox proportional hazards regression analysis was performed to evaluate the independent association between elevated PTH levels and long-term mortality. In the multivariable-adjusted model, the following variables remained independently associated with mortality: • older age • male sex • chronic kidney disease • solid malignancy • elevated PTH (>88 pg/mL). Importantly, elevated PTH remained independently associated with increased mortality risk after multivariable adjustment (HR 1.36, 95% CI 1.15–1.62, *p* < 0.001) (Table 3).

**Figure 2:**
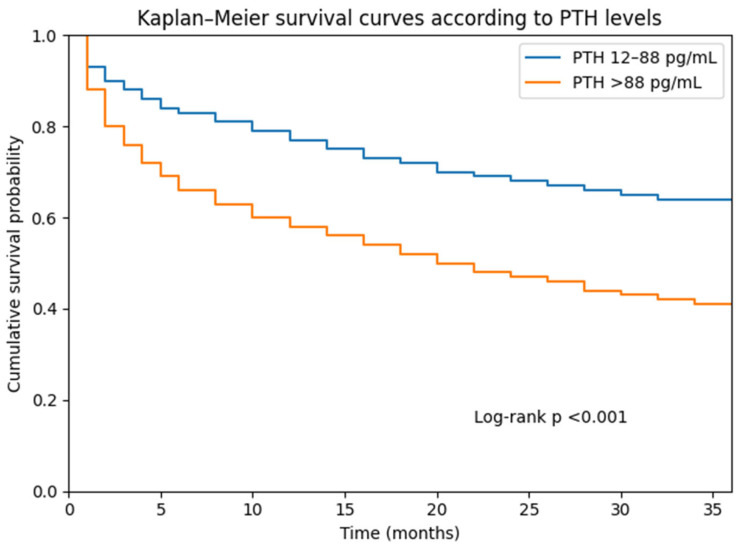
Kaplan–Meier survival curves according to PTH levels. Log-Rank (Mantel-Cox) Chi-square: 78.0, *p* < 0.001.

Kaplan–Meier survival curves demonstrated significantly lower cumulative survival in patients with elevated PTH levels compared with those with normal PTH levels (Figure 2). Tick marks on the survival curves represent censored observations, indicating patients who were alive at the end of follow-up or lost to follow-up. Censoring was handled according to standard survival analysis methodology.

## 4. Discussion

In this large real-world cohort of hospitalized internal medicine patients, elevated PTH levels were common and were associated with increased long-term mortality. Importantly, this association remained significant after adjustment for major demographic characteristics and comorbid conditions. These findings suggest that PTH may represent a clinically relevant biomarker reflecting disease burden in hospitalized patients.

Previous studies have demonstrated an association between elevated PTH and adverse outcomes in community-based cohorts and patients with chronic kidney disease. In the Hoorn Study, higher PTH levels were associated with increased all-cause and cardiovascular mortality [5]. Similarly, a meta-analysis including more than 31,616 participants confirmed that elevated PTH is linked to increased mortality risk [6]. Our findings are consistent with these observations and suggest that the prognostic relevance of PTH may extend beyond community and CKD populations to hospitalized patients with multimorbidity. One potential explanation for this association is the link between PTH and systemic inflammation. Experimental and clinical studies have shown that hyperparathyroidism is associated with elevated inflammatory cytokines, including interleukin-6 and tumor necrosis factor-α [7]. In population-based data, increasing PTH levels were positively correlated with inflammatory markers such as CRP and hematologic indices of systemic inflammation [8]. In our cohort, patients with elevated PTH demonstrated higher inflammatory markers and lower albumin levels, supporting the hypothesis that PTH may reflect inflammatory and catabolic states. Although prior studies have reported inconsistent findings regarding inflammatory markers in hyperparathyroidism [9], our results suggest that in hospitalized patients, elevated PTH may capture a broader inflammatory and metabolic burden.

Therefore, elevated PTH may represent not only a marker of mineral metabolism disturbance but also a surrogate indicator of systemic inflammation and physiological stress. Cardiovascular disease represents another plausible pathway linking PTH to mortality. Elevated PTH has been associated with vascular calcification, myocardial remodeling, arrhythmias, and left ventricular dysfunction [10,11]. In our cohort, ischemic heart disease was more prevalent among patients with elevated PTH levels and among those who died during follow-up. Prior longitudinal data have also demonstrated an association between PTH and cardiovascular mortality after adjustment for traditional risk factors [12]. Vitamin D deficiency may contribute to secondary hyperparathyroidism and has been linked to cardiovascular risk. However, whether correction of vitamin D deficiency effectively reduces PTH-related cardiovascular risk and mortality remains uncertain. Randomized controlled trials investigating vitamin D supplementation have produced inconsistent effects on cardiovascular outcomes and mortality, suggesting that the relationship between vitamin D, PTH, and adverse outcomes is likely complex and not solely mediated through correction of vitamin D deficiency.

Hypertension and chronic kidney disease are strongly linked to secondary hyperparathyroidism. Previous studies report hypertension prevalence between 40% and 65% in patients with hyperparathyroidism [13,14]. Extensive inpatient data have further demonstrated an independent association between primary hyperparathyroidism and hypertension [15]. In our study, hypertension and CKD were markedly more frequent in patients with elevated PTH, reinforcing the close relationship between PTH and cardiometabolic comorbidity. Interestingly, hypertension was not independently associated with mortality after multivariable adjustment, which may reflect the complex hemodynamic and multimorbid profile of older hospitalized patients. Consistent with this perspective, previous studies in very old populations, including centenarians, have demonstrated that elevated PTH levels are associated with increased all-cause mortality and may exhibit a J-shaped relationship with mortality risk [16]. Although age-specific reference intervals may be biologically relevant, we adjusted for age as a continuous variable in multivariable models. Our objective was to evaluate the prognostic performance of the routinely applied laboratory upper reference limit in real-world clinical practice rather than to redefine age-adjusted thresholds [17].

Importantly, PTH in this setting should be interpreted as a biomarker rather than a causal factor. In routine clinical practice, PTH is typically measured in patients with abnormalities in mineral metabolism, renal dysfunction, or suspected endocrine disorders. Therefore, elevated PTH likely reflects the combined effects of renal impairment, inflammation, malnutrition, and multimorbidity. This integrative role may explain why PTH retained prognostic significance even after adjustment for multiple comorbidities and laboratory parameters.

### 4.1. Strengths and Limitations

This study has several strengths. It includes a large real-world cohort of internal medicine inpatients with comprehensive clinical and laboratory data and long-term follow-up. However, several limitations should be acknowledged. First, the retrospective single-center design limits causal inference. Second, PTH measurements were performed based on clinical indication, which may introduce selection bias. Third, mortality data were derived from hospital records, and follow-up information was unavailable for a small subset of patients. Additionally, seasonal variation in vitamin D and PTH levels was not assessed, which may represent a potential source of biological variability. Finally, residual confounding cannot be fully excluded despite multivariable adjustment.

Future studies are needed to further clarify the mechanisms linking elevated PTH levels with adverse clinical outcomes in hospitalized populations. Prospective multicenter studies with standardized measurements of mineral metabolism markers may help determine whether PTH is merely a marker of disease severity or whether it plays a more direct role in the pathophysiology of adverse outcomes. In addition, evaluating whether integration of PTH into clinical risk prediction models improves prognostic accuracy in hospitalized patients represents an important direction for future research.

### 4.2. Clinical Implications

Our findings suggest that elevated PTH may help identify hospitalized patients at increased long-term mortality risk. Future prospective studies are needed to determine whether PTH is merely a marker of disease burden or a modifiable risk factor. The use of clinically measured PTH values reflecting real-world decision-making further enhances the external validity of the findings.

## 5. Conclusions

Elevated parathyroid hormone levels are common among hospitalized internal medicine patients and are associated with increased long-term mortality. In this real-world cohort, PTH appears to reflect the combined burden of renal dysfunction, inflammation, and multimorbidity. These findings suggest that PTH may serve as a useful biomarker for risk stratification in hospitalized populations. Prospective, multicenter studies are warranted to clarify the mechanisms underlying this association and determine the potential clinical utility of incorporating PTH into routine risk assessment.

## Figures and Tables

**Table 1:** Demographic characteristics, laboratory values and comorbid diseases of the patients.

Variable	PTH 12–88 pg/mL (*n* = 1028)	PTH > 88 pg/mL (*n* = 567)	*p*-Value
Demographic **characteristics**			
Age (**years**), mean ± SD	58 ± 18	68 ± 16	<0.001
**Female sex, *n* (%)**	493 (48%)	318 (56%)	0.02
**Laboratory findings**			
**Leukocyte (10^3^/µL), median (IQR)**	7.9 (5.9–10.7)	8.3 (6.1–11.3)	0.51
**Hemoglobin (g/dL), mean ± SD**	10.7 ± 2.8	9.8 ± 2.5	<0.001
**Platelet (10^3^/µL), mean ± SD**	237 ± 120	221 ± 100	0.01
**Neutrophil (10^3^/µL), median (IQR)**	5.1 (3.5–7.8)	5.6 (3.8–8.3)	0.03
**Lymphocyte (10^3^/µL), median (IQR)**	1.4 (0.9–2.0)	1.1 (0.7–1.7)	<0.001
**Procalcitonin (µg/L), median (IQR)**	0.22 (0.07–0.90)	0.30 (0.09–1.20)	0.13
**CRP (mg/L), median (IQR)**	32 (9–86)	41 (12–103)	0.01
**ALT (U/L), median (IQR)**	24 (15–45)	26 (16–48)	0.95
**GGT (U/L), median (IQR)**	35 (19–78)	37 (21–82)	0.29
**ALP (U/L), median (IQR)**	92 (68–134)	118 (84–168)	0.002
**Creatinine (mg/dL), median (IQR)**	0.9 (0.7–1.3)	2.1 (1.2–3.6)	<0.001
**LDL (mg/dL), mean ± SD**	94 ± 44	94 ± 47	0.74
**Triglycerides (mg/dL), median (IQR)**	125 (88–180)	121 (85–176)	0.84
**HbA1c (%), mean ± SD**	6.9 ± 2.5	6.5 ± 1.8	<0.001
**TSH (mU/L), median (IQR)**	1.8 (0.9–3.2)	2.0 (0.9–3.8)	0.09
**25(OH)D (ng/mL), mean ± SD**	13 ± 9	11 ± 9	0.01
**Albumin (g/L), mean ± SD**	33 ± 6	31 ± 5	<0.001
**Calcium (mg/dL), mean ± SD**	9.2 ± 0.6	8.9 ± 0.9	<0.001
**Phosphorus (mg/dL), mean ± SD**	3.3 ± 1.0	4.1 ± 1.5	<0.001
**Magnesium (mg/dL), mean ± SD**	1.9 ± 0.3	2.0 ± 0.4	0.01
**Comorbid conditions, *n* (%)**			
**Diabetes mellitus**	394 (38%)	232 (41%)	0.31
**Hypertension**	451 (44%)	371 (65%)	<0.001
**Ischemic heart disease**	235 (23%)	207 (37%)	<0.001
**Chronic kidney disease**	98 (10%)	261 (46%)	<0.001
**Thyroid disease**	88 (9%)	49 (9%)	0.42
**Solid malignancy**	102 (10%)	62 (11%)	0.54
**Hematologic malignancy**	24 (2%)	12 (2%)	0.78

CRP, C-reactive protein; ALT, alanine transaminase; GGT, gamma-glutamyl transferase; ALP, Alkaline phosphatase; LDL, low-density lipoprotein; HbA1c, hemoglobin A1C; TSH, thyroid-stimulating hormone; PTH, parathyroid hormone. Patients with hypothyroidism or hyperthyroidism were included.

**Table 2:** Demographic characteristics, laboratories and comorbid diseases by mortality.

Variable	Survived (*n* = 877)	Non-Survivors (*n* = 627)	*p*-Value
**Age (years), mean ± SD**	56 ± 18	72 ± 12	<0.001
**Female sex, *n* (%)**	464 (53%)	301 (48%)	0.06
**Leukocyte (10^3^/µL), median (IQR)**	7.7 (5.8–10.2)	8.9 (6.4–11.9)	0.01
**Hemoglobin (g/dL), mean ± SD**	10.7 ± 2.8	9.8 ± 2.8	<0.001
**Platelet (10^3^/µL), mean ± SD**	238 ± 109	224 ± 115	0.02
**Neutrophil (10^3^/µL), median (IQR)**	4.9 (3.3–7.2)	6.0 (4.2–8.9)	<0.001
**Lymphocyte (10^3^/µL), median (IQR)**	1.4 (0.9–2.1)	1.0 (0.7–1.5)	<0.001
**Procalcitonin (µg/L), median (IQR)**	0.20 (0.06–0.80)	0.28 (0.09–1.10)	0.97
**CRP (mg/L), median (IQR)**	28 (8–71)	55 (17–120)	<0.001
**Creatinine (mg/dL), median (IQR)**	0.9 (0.7–1.4)	1.5 (0.9–2.7)	<0.001
**PTH (pg/mL), median (IQR)**	63 (39–116)	104 (56–210)	<0.001
**Albumin (g/L), mean ± SD**	34 ± 5	29 ± 5	<0.001
**Diabetes mellitus**	335 (38%)	270 (43%)	0.06
**Hypertension**	416 (47%)	379 (60%)	<0.001
**Ischemic heart disease**	205 (23%)	224 (36%)	<0.001
**Chronic kidney disease**	134 (15%)	206 (33%)	<0.001
**Solid malignancy**	33 (4%)	128 (20%)	<0.001

CRP, C-reactive protein; PTH, Parathyroid hormone.

**Table 3:** Multivariate analysis of factors affecting mortality.

Variable	*p*-Value	Hazard Ratio	95% CI
**Model 1**			
Age	<0.001	1.05	1.04–1.06
Male sex	0.04	1.19	1.01–1.40
Hypertension	0.002	0.76	0.64–0.91
Chronic kidney disease	<0.001	1.43	1.18–1.73
Solid malignancy	<0.001	3.35	2.75–4.08
PTH > 88 pg/mL	<0.001	1.37	1.15–1.64
**Model 2 (multivariable)**			
Age	<0.001	1.05	1.04–1.05
Male sex	0.03	1.19	1.01–1.40
Hypertension	0.01	0.78	0.66–0.93
Chronic kidney disease	<0.001	1.46	1.21–1.76
Solid malignancy	<0.001	3.29	2.71–4.01
PTH > 88 pg/mL	<0.001	1.36	1.15–1.62

PTH: Parathyroid Hormone. Model 1: Full Cox regression model. Model 2: Reduced multivariable model.

## Data Availability

The datasets generated and analyzed during the current study are available from the corresponding author upon reasonable request.
